# 202. Association of Neutrophil - Lymphocyte Ratio with Bacteremia and In-Hospital Mortality in Sepsis Patients: A Retrospective Multicenter Study

**DOI:** 10.1093/ofid/ofad500.275

**Published:** 2023-11-27

**Authors:** Shahin Isha, Sadhana Jonna, Lekhya Raavi, Anna Jenkins, Abby J Hanson, Parthkumar Satashia, Siva Naga S Yarrarapu, Austin Govero, Michael F Harrison, Hassan Z Baig, Sean M Caples, Ami A Grek, Michael R Vizzini, Syed A Khan, Katherine Heise, Hiroshi Sekiguchi, Warren Cantrell, Jeffrey D Smith, Karthik Gnanapandithan, Kristine M Thompson, Charles G Graham, Jed C Cowdell, Aleksandra Murawska Baptista, Anna B Shapiro, Anirban Bhattacharyya, Sanjay Chaudhary, Sean Kiley, Pramod K Guru, Claudia Libertin, Pablo Moreno Franco, Devang K Sanghavi

**Affiliations:** Ascension Saint Joseph Hospital, Chicago, Chicago, Illinois; Mayo Clinic, Florida, Jacksonville, Florida; Mayo Clinic, Florida, Jacksonville, Florida; Mayo Clinic, Florida, Jacksonville, Florida; Mayo Clinic, Florida, Jacksonville, Florida; Mayo Clinic, Florida, Jacksonville, Florida; Monmouth Medical Center, Monmouth, New Jersey; Mayo Clinic, Florida, Jacksonville, Florida; Mayo Clinic, Florida, Jacksonville, Florida; Mayo Clinic, Florida, Jacksonville, Florida; Mayo Clinic, Rochester, Rochester, Minnesota; Mayo Clinic, Florida, Jacksonville, Florida; Mayo Clinic, Florida, Jacksonville, Florida; Mayo Clinic Health System in Mankato, Mankato, Minnesota; Mayo Clinic, Florida, Jacksonville, Florida; Mayo Clinic, Arizona, Jacksonville, Florida; Mayo Clinic, Florida, Jacksonville, Florida; Mayo Clinic, Florida, Jacksonville, Florida; Mayo Clinic, Florida, Jacksonville, Florida; Mayo Clinic, Florida, Jacksonville, Florida; Mayo Clinic, Florida, Jacksonville, Florida; Mayo Clinic, Florida, Jacksonville, Florida; Mayo Clinic, Florida, Jacksonville, Florida; Mayo Clinic, Florida, Jacksonville, Florida; Mayo Clinic, Florida, Jacksonville, Florida; Mayo Clinic, Florida, Jacksonville, Florida; Mayo Clinic, Florida, Jacksonville, Florida; Mayo Clinic, Florida, Jacksonville, Florida; Mayo Clinic, Florida, Jacksonville, Florida; Mayo Clinic, Florida, Jacksonville, Florida; Mayo Clinic, Florida, Jacksonville, Florida

## Abstract

**Background:**

Early diagnosis, prognostication, and treatment initiation are the cornerstones of sepsis management. There remains an immense interest in exploring the diagnostic and prognostic roles of neutrophil-lymphocyte ratio (NLR) in the sepsis population.

**Methods:**

We performed a retrospective multi-center observational study including patients admitted to different Mayo Clinic sites with a diagnosis of sepsis between October 2018 and August 2022. Patients were excluded if they were aged < 18 years, lacked research authorization, or had missing Neutrophil or Lymphocyte count at admission. Data were collected from an existing sepsis database and Mayo Data Explorer (MDE). Categorical variables were summarized as percentages and continuous variables were summarized as medians. Chi-square test for significance, independent sample t-test, and multivariate Cox Proportional Hazard models were performed on IBM SPSS Statistics v28.0.

**Results:**

Our study cohort consisted of 13968 patients, of which the majority were males (55.9%), Caucasian (91.6%), and non-Hispanic or Latino (94.3%). The median age of the cohort was 71 (60, 80) years, and the median BMI was 27.7 (23.4, 33.3) kg/m2.

Among all sepsis patients, 6.0% had a positive blood culture and the most common organisms were E. coli (26.5%), Klebsiella sp. (15.6%), Streptococcus sp. (12.8%) and Staphylococcus aureus (9.9%). 13131 patients (94.1%) survived the hospital stay and 837 (5.9%) did not. There was no difference in mortality based on blood culture positivity status (6.0% vs 6.6%, p=0.41).

However, the median NLR was higher among patients with a positive blood culture (16.4 vs. 12.1, p< 0.001) and among non-survivors (14.3 vs 12.2, p< 0.001). NLR did not significantly vary based on the type of organism growing in the blood culture. A multivariate model revealed NLR as an independent predictor of mortality (HR 1.003, 95% CI: 1.000-1.006, p=0.029) after adjusting for comorbidities, baseline clinical and laboratory variables. (Tables 1 & 2)

**Figure:**
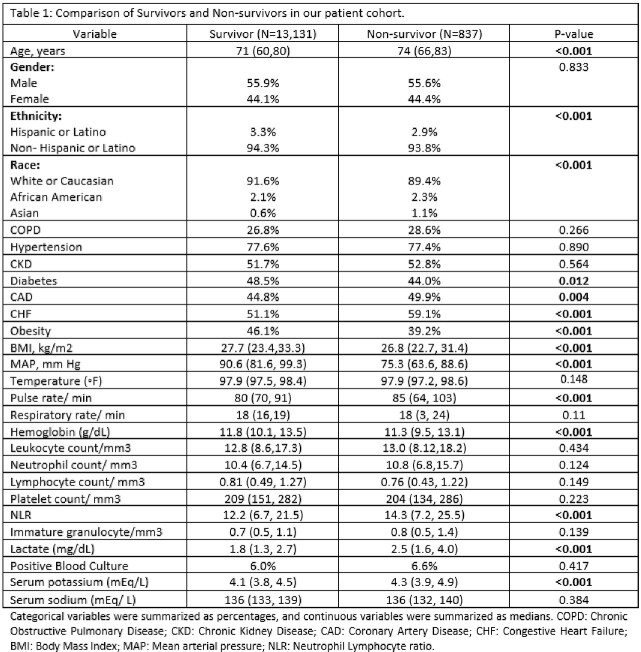

**Conclusion:**

Our retrospective multicentric study showed that the Neutrophil-Lymphocyte ratio at admission was higher among bacteremic sepsis patients and non-survivors. Further prospective studies are needed to explore its diagnostic and prognostic utility.

**Disclosures:**

**All Authors**: No reported disclosures

